# *Salvia miltiorrhiza* and* Carthamus tinctorius* Extract Prevents Cardiac Fibrosis and Dysfunction after Myocardial Infarction by Epigenetically Inhibiting Smad3 Expression

**DOI:** 10.1155/2019/6479136

**Published:** 2019-05-30

**Authors:** Jing Yang, Bo Wang, Na Li, Qingqing Zhou, Wenhui Zhou, Zhenzhen Zhan

**Affiliations:** ^1^Department of Cardiology, Shanghai Institute of Cardiovascular Diseases, Zhongshan Hospital, Fudan University, Shanghai 200032, China; ^2^Institute of Heart Failure, Shanghai East Hospital, Tongji University School of Medicine, Shanghai 200120, China; ^3^Department of Cardiology, Changzheng Hospital, Second Military Medical University, Shanghai 200003, China; ^4^Shanghai Fourth People's Hospital, Tongji University School of Medicine, Shanghai 200081, China

## Abstract

The incidence of cardiac dysfunction after myocardial infarction (MI) continues to increase despite advances in treatment. Excessive myocardial fibrosis plays a vital role in the development of adverse cardiac remodeling and deterioration of cardiac function. Understanding the molecular and cellular mechanism of the fibrosis process and developing effective therapeutics are of great importance.* Salvia miltiorrhiza* and* Carthamus tinctorius* extract (SCE) is indicated for angina pectoris and other ischemic cardiovascular diseases in China. SCE has been shown to inhibit the platelet activation and aggregation, ameliorate ROS-induced myocardial necrosis by inhibiting mitochondrial permeability transition pore opening, and promote angiogenesis by upregulating the expression of vascular endothelial growth factor (VEGF). However, whether SCE has effect on cardiac fibrosis after MI is not fully clear. Here, a mouse model of MI was established to observe the effect of SCE upon survival, cardiac function, myocardial fibrosis, and inflammation. Quantitative PCR and western blot assays were used to determine the expression of genes related to transforming growth factor-*β* (TGF-*β*) cascade and inflammatory responses* in vivo*. Additionally, the effects of SCE upon the collagen production, TGF-*β*/Smad3 (SMAD family member 3) signaling, and the levels of histone methylation in primary cardiac fibroblasts were detected. We found that SCE treatment significantly improved survival and left ventricular function in mice after MI. Inhibition of inflammation and fibrosis, as well as decreased expression of Smad3, was observed with SCE treatment. In TGF-*β*-stimulated cardiac fibroblasts, SCE significantly decreased the expression of collagen, *α*-smooth muscle actin (*α*-SMA), and Smad3. Furthermore, SCE treatment downregulated the levels of H3K4 trimethylation (H3K4me3) and H3K36 trimethylation (H3K36me3) at the* Smad3* promoter region of cardiac fibroblasts, leading to inhibition of* Smad3* transcription. Our findings suggested that SCE prevents myocardial fibrosis and adverse remodeling after MI with a novel mechanism of suppressing histone methylation of the* Smad3* promoter and its transcription.

## 1. Introduction

Heart failure following acute myocardial infarction remains a leading cause of global morbidity and mortality [1]. In recent decades, with the advances in reperfusion therapy, guideline-recommended medications, and devices for ventricular unloading, in-hospital mortality has decreased substantially to less than 5% [2]. However, due to the combination of reduced short-term mortality and insufficient long-term treatment to survivors after MI, the incidence of heart failure is gradually increasing [3, 4].

Myocardial fibrosis, characterized as deposition of collagen and accumulation of cardiac fibroblasts (CFs), is the pathological hallmark in left ventricular (LV) remodeling. The contribution of fibrosis to infarcted hearts varies depending on specific phases and sites after abrupt total occlusion of a coronary artery. Reparative fibrosis in the infarcted area triggered by myocardial loss is critical for wound healing. However, excessive fibrosis in the extracellular matrix (ECM) accompanied by overactivated myofibroblasts increases myocardial stiffness and subsequently remodels the ventricular structure, resulting in systolic and diastolic dysfunction, which eventually leads to heart failure [5]. Currently, no pharmaceutical agent can effectively control the fibrotic response to preserve left ventricular function and prevent heart failure. Therefore, a better understanding of the molecular and cellular mechanisms underlying the fibrosis process and the discovery of alternative therapeutic targets against detrimental fibrosis are of great importance for clinical practice.

Cardiac fibroblasts, as the dominant cell type in the adult heart, contribute predominantly to myocardial fibrosis after MI with differentiation into myofibroblasts [6]. Among the cytokines involved in the process of repair and remodeling, transforming growth factor-*β* (TGF-*β*) plays a central role in the promotion of fibroblast proliferation, myofibroblast differentiation, and ECM production with the expression of *α*-smooth muscle actin (*α*-SMA) [7]. In fibroblasts, activation of the TGF-*β* receptor causes the transcription factors SMAD family members 2/3 (Smad2/3) to complex with Smad4 in the nucleus and subsequently promotes expression of fibrosis-related genes [8]. Although some signaling proteins have been found to modulate fibrotic responses, the epigenetic regulation mechanism of myocardial fibrosis after MI needs further investigation [9].

*Salvia miltiorrhiza* and* Carthamus tinctorius* extract (SCE) is a standardized medication prepared from two Chinese herbs:* Salviae miltiorrhizae* (Danshen in Chinese) and* Flos carthami* (Honghua in Chinese). The main pharmacological active components of SCE are as follows: phenolic acids, diterpenes, and flavonoids, such as salvianolic acid B, danshensu, tanshinone IIA, and hydroxysafflor yellow A. SCE is widely used in patients with coronary heart disease for relieving angina [10]. Administration of SCE as a supplementary therapy appears to show benefit to the patients during the acute phase of MI [11]. Previous researches have suggested the therapeutic effect of SCE that may contribute to the infarct healing, including inhibition of the platelet activation and aggregation, anti-inflammation, amelioration of ROS-induced myocardial necrosis, regulation of arginine vasopressin expression and secretion, and promotion of angiogenesis [12–17]. In addition, SCE has been documented to attenuate cardiac hypertrophy both* in vivo* and* in vitro*, which is considered as compensation for myocardial loss [18, 19]. However, the effect of SCE on adverse myocardial fibrosis following MI remains unclear.

Here we found that SCE administration* in vivo* significantly increased the survival, improved cardiac function, and inhibited myocardial tissue inflammation in mice after MI in comparison with the control group. SCE treatment also decreased the levels of H3K4 trimethylation (H3K4me3) and H3K36 trimethylation (H3K36me3) at the* Smad3* promoter and its transcription in cardiac fibroblasts, which contributed to the suppressed expression of fibrosis-related genes and the attenuated myocardial fibrosis in mice after MI.

## 2. Materials and Methods

### 2.1. Mouse Model of MI

Wild-type C57BL/6 mice were purchased from Joint Ventures Sipper BK Experimental Animal Company (Shanghai, China). All animal experiments were conducted according to the National Institute of Health Guide for the Care and Use of Laboratory Animals, which was approved by the Scientific Investigation Board of Tongji University, Shanghai. 8–10-week-old male mice received MI operation by permanent left anterior descending (LAD) coronary artery ligation. Briefly, mice were anesthetized with isoflurane (induced at 4%, maintained at 1.5%), intubated, and ventilated with a small animal ventilator. After left thoracotomy, the LAD was permanently ligated 2 mm below the origin of the LAD using a 6-0 suture. Significant blanching at the ischemic area was indicative of successful modeling. The chest was closed with a 5-0 suture. Once spontaneous respiration resumed, the endotracheal tube was removed. Mice were placed on a warm pad maintained at 37°C and were closely supervised until full consciousness was regained. Sham surgery was performed exactly as above without coronary artery ligation. MI mice were randomized and assigned to the SCE group and control group. Mice in the SCE group received intraperitoneal SCE administration of 3 *μ*l per g body weight per day; mice in the control group received normal saline intraperitoneally. Echocardiography assays were performed at 1, 2, and 3 weeks after MI. At endpoint, mice were sacrificed for the collection of heart samples.

### 2.2. Isolation and Culture of Neonatal Cardiac Fibroblasts

Hearts of 1–3-day-old neonatal mice were excised and rinsed in cold Hank's balanced salt solution (Gibco). Then heart tissues were minced and digested with type II collagenase (100 U/ml) (Thermo) and pancreatin (0.25%) (Sigma-Aldrich) by rocking at 37°C for 15 min. After discarding supernatant, the partially digested heart fragments continued to be digested in the above digestion solution by pipetting for 12-15 times and rocking at 37°C for 15 min. The collagenase medium from the second digestion containing CFs was mixed with 10% horse serum (Gibco) and centrifuged for 5 min at 1500g and then resuspended in DMEM with 10% fetal bovine serum (Gibco). The digestion was repeated for 2-3 times until the digestion solution became clear. Cells were plated in 60 mm dishes (Corning) and allowed to attach for 1 h before the first media change. Fibroblasts were trypsinized and passaged as required based on cellular confluence.

### 2.3. Reagents and Antibodies

SCE (merchandised as Danhong Injection in China) was provided by Heze Buchang Pharmaceutical Co., Ltd. of China (code number approved by National Medical Products Administration Z20026866; Batch number 16081052). According to the statement of National Medical Products Administration standard, the quality control standard of SCE is that the total amount of protocatechuic aldehyde (molecular formula is C7H6O3) and danshensu (molecular formula is C9H10O5) should not be lower than 0.5 mg analyzed by high-performance liquid chromatography (HPLC) as a reference in 1 ml injection. In addition, the total flavonoids determined by visible spectrophotometry should not be lower than 5.0 mg/ml against rutin (molecular formula: C27H30O16). HPLC fingerprinting of SCE also has been reported. The quality control of SCE was performed as previously described [19]. Recombinant mouse TGF-*β*1 protein (7666-MB) was from R&D Systems. Antibody against collagen type I (600-401-103) was from Rockland Immunochemicals Inc. Antibody against collagen III alpha 1/COL3A1 (NB600-594) was from Novus Biologicals. Antibodies against *α*-SMA antibody (ab5694), Smad3 (ab40854), and Smad7 (ab216428) were from Abcam. Antibodies against Smad2 (5339) and *β*-actin (3700) were from Cell Signaling Technology. Antibodies against H3K4me3 (17-614) and H3K36me3 (17-10032) were from Merck Millipore. Chemicals frequently used in our laboratory were purchased from Sigma-Aldrich.

### 2.4. Echocardiography

Transthoracic echocardiography was carried out with a Vevo 2100 instrument (VisualSonics) equipped with an MS-400 imaging transducer. Mice were kept awake, without anesthesia, during the echocardiographic examination to minimize data deviation, and the heart rate was maintained at 550-650 bpm in all mice. M-mode tracings were recorded through the anterior and posterior LV walls at the papillary muscle level to measure LV end-diastolic dimension (LVEDD) and LV end-systolic dimension (LVESD). LV fractional shortening (LVFS) was calculated according to the following formula: FS = [(LVEDD-LVESD)/LVEDD] × 100. End-diastolic volume (EDV), end-systolic volume (ESV), and ejection fraction (LVEF) were calculated by using the spherical formula.

### 2.5. Histological Analysis

The myocardial sample was fixed with 4% paraformaldehyde for 24 h, dehydrated through increasing concentrations of ethanol, and then embedded in paraffin. LV sections (5 *μ*m) were stained with Masson's trichrome (Sigma-Aldrich) following the manufacturers' instructions. In brief, nuclei are stained with Weigert's iron hematoxylin, and cytoplasm and muscle are then stained with Beibrich scarlet-acid fuchsin. After treatment with phosphotungstic and phosphomolybdic acid, collagen is demonstrated by staining with aniline blue. Rinsing in acetic acid after staining renders the shades of color more delicate and transparent. Images were recorded by A Nikon Eclipse 80i microscope and NIS Elements software. Dedicated software was used to analyze the area of fibrosis in the border zone on Masson's trichrome-stained sections. Percent scar circumference was determined as total infarct circumference divided by total LV circumference × 100.

### 2.6. RNA Extraction and Reverse Transcription Quantitative PCR (RT-qPCR)

Total RNA from the heart tissues and cultured cells was extracted using TRIzol reagent (Invitrogen) according to the manufacturer's instructions. RNA was reverse-transcribed to cDNA using the first-strand cDNA synthesis kit (TOYOBO). cDNA was used for quantitative PCR analysis with Applied Biosystems 7500 System (Thermo Fisher). Data were normalized by the level of glyceraldehyde-3-phosphate dehydrogenase (GAPDH) expression in each sample. The primer pairs used were listed in Supplemental Table1.

### 2.7. Western Blot Analysis

Samples from heart tissue or cultured cells were homogenized in lysis buffer containing a proteinase and phosphatase inhibitor cocktail. The western blot assay was performed using standard techniques with an SDS-PAGE electrophoresis system. Collagen I, collagen III, *α*-SMA, Smad3, Smad2, and Smad7 were detected with specific antibodies. *β*-Actin level was used as an internal control.

### 2.8. Chromatin Immunoprecipitation (ChIP) Assay

Cells were cross-linked with 1% (vol/vol) methanol-free formaldehyde for 10 minutes and stopped with 125 mM glycine. Nuclei were isolated by resuspending the cells in a swelling buffer containing 5 mM PIPES pH 8.0, 85 mM KCl, 1% NP-40, and a complete protease inhibitor for 20 min at 4°C. The isolated nuclei were resuspended in nuclei lysis buffer (50 mM Tris pH 8.0, 10 mM EDTA, 1% SDS) and sonicated using a Bioruptor Sonicator (Diagenode). The samples were immunoprecipitated with 2-4 *μ*g of H3K4me3 and H3K36me3 antibodies (Millipore) overnight at 4°C. Protein G agarose beads were used and incubated for 15 min, and the immune precipitates were washed twice with dialysis buffer (50 mM Tris pH 8.0, 2 mM EDTA, 0.2% Sarkosyl) and four times with IP wash buffer (100 mM Tris pH 8.0 or pH9.0, 500 mM LiCl, 1% NP-40, and 1% deoxycholic acid sodium salt). Bound DNA was eluted using fresh 50 mM NaHCO3 and 1% SDS, reverse cross-linked, and purified using a PCR purification kit (Qiagen). Fold enrichment was quantified using quantitative PCR, and data were normalized by input DNA for each sample. The primers used for* Smad3* promoter (-208 to -8) detection were as follows: 5′-GCTGCACGCGGAGCTGCGTGAAA-3′ (sense) and 5′-GGAAAGTCCAACCCCCAGCCAAA-3′ (antisense).

### 2.9. Statistical Analysis

The statistical significance of comparisons between 2 groups was determined with an unpaired Student's* t*-test. For comparison of more than two groups, one-way analysis of variance (ANOVA) with LSD* t*-test was used. Survival rate was analyzed by the Kaplan-Meier method, and differences between groups were determined by a log-rank (Mantel-Cox) test. Statistical analyses were performed by SPSS (version 22.0) statistical software. Differences with* P *< 0.05 were considered significant.

## 3. Results

### 3.1. SCE Attenuates Cardiac Dysfunction and Myocardial Fibrosis after MI

**Figure 1:**
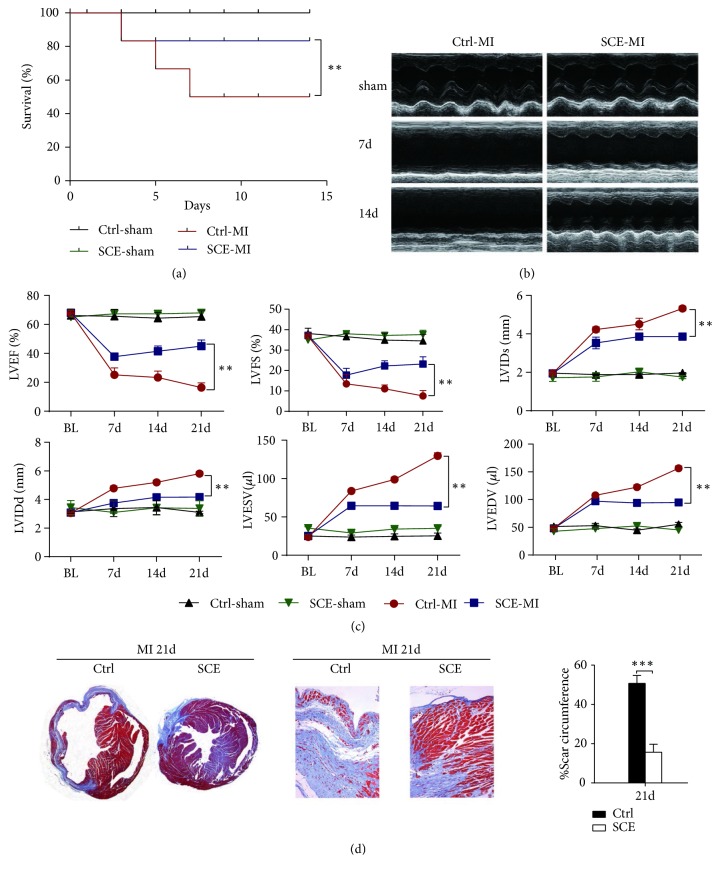
SCE treatment improves survival and cardiac function and reduces fibrosis size in mice after MI. (a) Survival curve for MI mice treated with SCE (SCE + MI group, n = 17 mice; Ctrl + MI group, n = 20 mice). ^*∗∗*^*P* < 0.01, Student's* t*-test. (b) Representative M-mode images 21 days after MI. (c) Left ventricular ejection fraction (LVEF), left ventricular fractional shortening (LVFS), left ventricular internal dimension at end-systole (LVIDs), left ventricular internal dimension at end-diastole (LVIDd), left ventricular end-systolic volume (LVESV), and left ventricular end-diastolic volume (LVEDV) were quantified via echocardiography. Data represent the mean ± SEM (n = 10 mice per group). ^*∗∗*^*P* < 0.01, Student's* t*-test. (d) Left, representative Masson's trichrome staining of the coronal planes and the border zone 21 days following MI. Right, quantification of scar circumference in both the SCE group and the control group 21 days following MI. Data represent the mean ± SEM (n = 5 mice per group). ^*∗∗∗*^*P* < 0.001, Student's* t*-test.

We first examined the effect of SCE administration* in vivo* on survival, cardiac function, and myocardial fibrosis after MI. Administration of SCE markedly decreased mortality compared with the control group (Figure 1(a)). The disparity in mortality was not observed between the two groups until 5 days, which is the fibrotic phase succeeding the resolved inflammatory phase. Autopsy demonstrated that LV rupture and pulmonary edema were the two major causes of death after MI. Therefore, the reduction of mortality mediated by SCE might be related to the promotion of wound repair as well as depression of fibrosis. Furthermore, mice with SCE administration had improved cardiac function as shown by the significantly increased LVEF and LVFS but decreased LVIDd, LVIDs, LVEDV, and LVESV compared with those of the control mice (Figures 1(b) and 1(c)). Consistent with echocardiographic results, SCE treatment alleviated collagen deposition and myocardium loss in the amplified infarct border zones (Figure 1(d)). The scar tissue percent circumferences at day 21 after MI were significantly decreased in SCE-treated mice than those in control mice (Figure 1(d)). These data indicated that SCE administration* in vivo* prevented cardiac dysfunction and myocardial fibrosis after MI.

### 3.2. SCE Suppresses MI-Induced Inflammation in Heart Tissue

**Figure 2:**
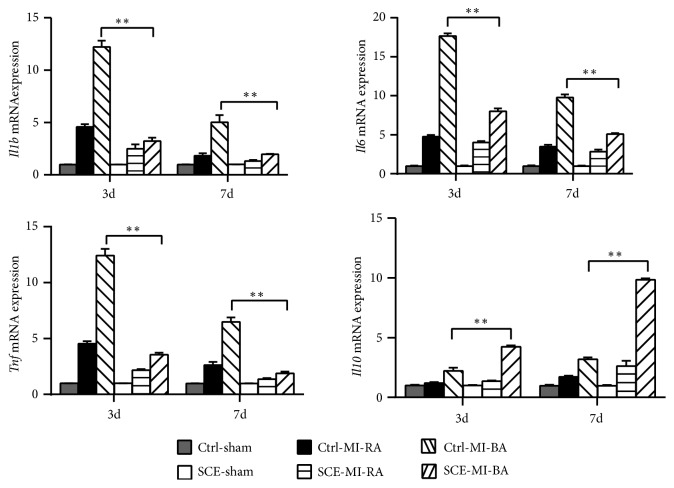
SCE suppresses MI-induced inflammatory responses in heart tissue. RT-qPCR analysis of* Il1b*,* Il6*,* Tnf*, and* Il10* mRNA in heart tissues from sham group, remote area (RA), or infarct border area (BA) from mice with SCE administration (SCE) or control mice (Ctrl) (n = 3 mice per group) at the indicated days after MI. Expression levels were shown as relative fold values compared with the sham group (calculated as 1 for each gene at the indicated days) after normalization to* GAPDH*. Data are from three independent experiments (mean ± SEM). ^*∗∗*^*P* < 0.01, ANOVA with LSD* t*-test.

Inflammation is another main cause of myocardial necrosis beyond ischemic injury and subsequent cardiac remodeling. To confirm the effect of SCE on inflammation, RT-qPCR was performed to measure the expression of inflammatory cytokines in the early phase after MI. At either day 3 or day 7, the expressions of proinflammatory cytokines, such as* interleukin 1 beta* (*Il1b*),* tumor necrosis factor* (*Tnf*), and* interleukin 6* (*Il6*), in the border area of cardiac tissue in the SCE group were significantly lower than those in the control group, while the expression of anti-inflammatory cytokine* interleukin 10* (*Il10*) was significantly higher in the SCE group (Figure 2). These findings suggested that the role of SCE in suppressing inflammation was correlated with its beneficial effect on cardiac fibrosis.

### 3.3. SCE Attenuates Myocardial Fibrosis after MI in Mice

**Figure 3:**
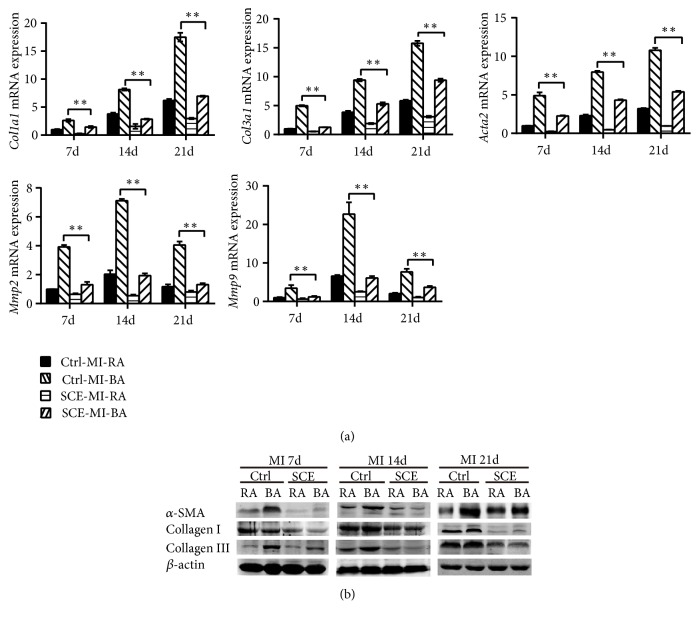
SCE attenuates myocardial fibrosis in heart tissue after MI. (a) RT-qPCR analysis for mRNA expression of* collagen type I alpha 1 *(*Col1a1*),* collagen type III alpha 1 *(*Col3a1*),* actin, alpha 2 smooth muscle *(*Acta2*),* Mmp2*, and* Mmp9* in the border area (BA) and remote area (RA) 7 days, 14 days, and 21 days after MI. (b) Immunoblot analysis of protein expressions of *α*-SMA, collagen I, and collagen III in RA and BA in the heart tissues 7 days, 14 days, and 21 days after MI. Data are from three independent experiments ((a), mean ± SEM) or are representative of three independent experiments with similar results (b). ^*∗*^*P* < 0.05 and ^*∗∗*^*P* < 0.01, ANOVA with LSD* t*-test.

Transformation and activation of myofibroblasts, characterized by expression of *α*-SMA and production of ECM components, is a hallmark event in fibrotic remodeling. We further determined the effect of SCE on the expression level of fibrosis-related genes* in vivo*. The expressions of collagen I, collagen III, and *α*-SMA in the border areas were observed to be significantly higher than those in the remote regions, and the expression increased gradually from the early phase to the late phase after MI. Compared with the control group, SCE administration reduced the expressions of collagen I, collagen III, and *α*-SMA in both the mRNA and protein levels in the remote myocardium at all time points (Figures 3(a) and 3(b)). The excessive secretion of metal matrix proteins (MMPs) promoting degradation of the matrix was closely related to ventricular enlargement and dysfunction. SCE administration also decreased the expression levels of* Mmp2* and* Mmp9* genes in the remote areas (Figure 3(a)). These data indicated that SCE attenuated myocardial fibrosis in mice after MI.

### 3.4. SCE Decreases Smad3 Expression after MI in Mice

**Figure 4:**
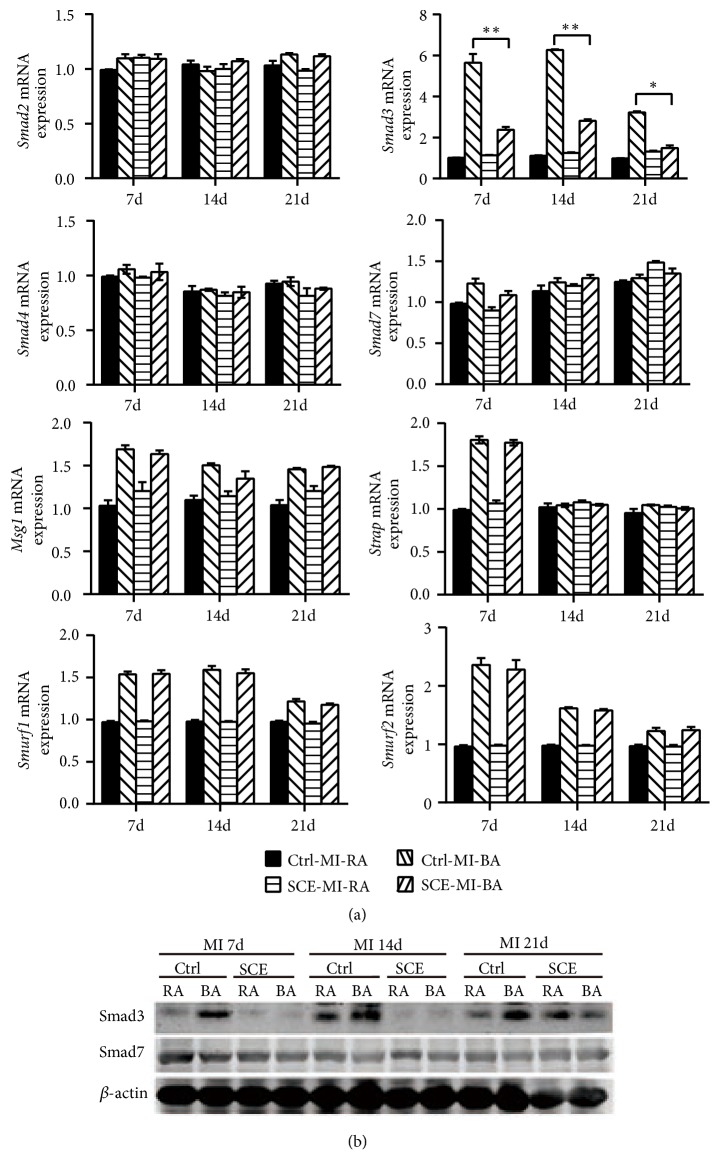
SCE decreases Smad3 expression in heart tissue after MI. (a) RT-qPCR analysis for mRNA expression of* Smad2*,* Smad3, Smad4*,* Smad7*,* Msg1*,* Strap*,* Smurf1*, and* Smurf2* in the border area (BA) and remote area (RA) of the infarcted hearts which were excised 7 days, 14 days, and 21days after MI. (b) Immunoblot analysis of Smad3 and Smad7 protein expression in the BA and RA of the infarcted hearts excised 7 days, 14 days, and 21days after MI. Data are from three independent experiments ((a), mean ± SEM) or are representative of three independent experiments with similar results (b). ^*∗*^*P* < 0.05 and ^*∗∗*^*P* < 0.01, ANOVA with LSD* t*-test.

Activation of the TGF-*β*-dependent signaling pathway plays a central role in the fibrosis process after MI. We next explored the signaling molecules that mediated the inhibitive effect of SCE on fibrosis. SCE administration had no effect on the expression of* Smad2*,* Smad4*,* Smad7*,* Melanocyte-specific gene 1 *(*Msg1*)*, serine/threonine kinase receptor associated protein *(*Strap*)*, SMAD specific E3 ubiquitin protein ligase 1 *(*Smurf1*)*, SMAD specific E3 ubiquitin protein ligase 2 *(*Smurf2*)*, Transducer of ErbB2 *(*Tob*)*, Ski-related novel protein N *(*Snon*)*, myelocytomatosis oncogene *(*C-Myc*)*, Ecotropic Viral Integration site 1 *(*Evi1*)*, zinc finger E-box binding homeobox 2 *(*Zeb2*)*, and CREB binding protein *(*Cbp*) (Figure 4(a) and FigureS1 in the Supplementary Material) in cardiac tissue after MI. Only Smad3 expression in cardiac tissue was significantly decreased by SCE administration (Figure 4), suggesting that SCE attenuated myocardial fibrosis after MI by inhibiting the expression of Smad3.

### 3.5. SCE Ameliorates Fibrotic Responses in CFs by Inhibiting TGF-*β*/Smad3 Signaling Activation

**Figure 5:**
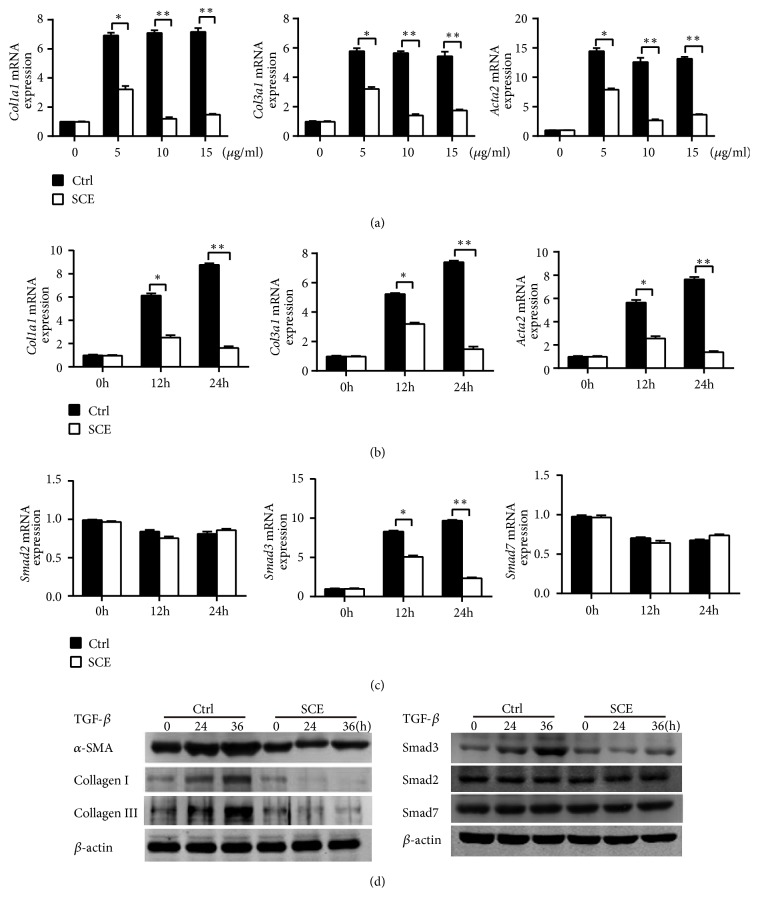
SCE attenuates fibrotic responses in CFs by inhibiting TGF-*β*/Smad3 signaling activation. (a) RT-qPCR analysis of mRNA expression for genes of* Col1a1*,* Col3a1*, and* Acta2* in cardiac fibroblasts (CFs) pretreated with different concentrations of SCE (5 *μ*l/ml, 10 *μ*l/ml, and 15 *μ*l/ml) followed by TGF-*β* stimulation (20 ng/ml) for 24 h. (b) RT-qPCR analysis of mRNA expression for genes of* Col1a1*,* Col3a1,* and* Acta2* in CFs pretreated with SCE (10 *μ*l/ml) followed by TGF-*β* stimulation (20 ng/ml) for 12 h and 24 h. (c) RT-qPCR analysis of mRNA expression for genes of* Smad2*,* Smad3,* and* Smad7* in CFs pretreated with SCE (10 *μ*l/ml) followed by TGF-*β* stimulation (20 ng/ml) for 12 h and 24 h. (d) Immunoblot analysis of expression of Smad2, Smad3, and Smad7 in CFs pretreated with SCE (10 *μ*l/ml) followed by TGF-*β* stimulation (20 ng/ml) for 24 h and 36 h. Data are from three independent experiments ((a)–(c), mean ± SEM) or are representative of three independent experiments with similar results (d). ^*∗*^*P* < 0.05 and ^*∗∗*^*P* < 0.01, Student's* t*-test.

CFs, as the main cellular effector of collagen formation, can activate and transform into myofibroblasts, which play a crucial role in cardiac fibrosis. We then investigated the effect of SCE treatment on the expression of fibrosis-related genes in TGF-*β*-stimulated CFs. The treatment of SCE at concentrations of 5, 10, or 15 *μ*l/ml did not induce apoptosis of CFs (FigureS2 in the Supplementary Material). SCE treatment decreased the mRNA expression levels of* Col1a1*,* Col3a1,* and* Acta2 *in CFs induced by TGF-*β*, most significantly at a concentration of 10 *μ*l/ml (Figure 5(a)). SCE also inhibited the expression of collagen I, collagen III, and *α*-SMA at the mRNA and protein levels in TGF-*β*-stimulated CFs in a time-dependent manner (Figures 5(b) and 5(d)). The expressions of* Mmp2*,* Mmp9*,* tenascin C *(*Tnc*)*, fibronectin 1 *(*Fn1*)*, transgelin *(*Tagln*), and* connective tissue growth factor* (*Ctgf*) were also decreased upon SCE treatment in TGF-*β*-stimulated CFs (FigureS3 in the Supplementary Material). We further found that the SCE treatment suppressed the mRNA and protein expression level of Smad3, but not Smad2 or Smad7, in TGF-*β*-stimulated CFs (Figures 5(c) and 5(d)). These data indicated that SCE selectively decreased Smad3 expression and then inhibited TGF-*β*/Smad3 signaling activation, which ameliorated fibrotic responses in CFs.

### 3.6. SCE Decreases Histone Methylation at the Smad3 Promoter

**Figure 6:**
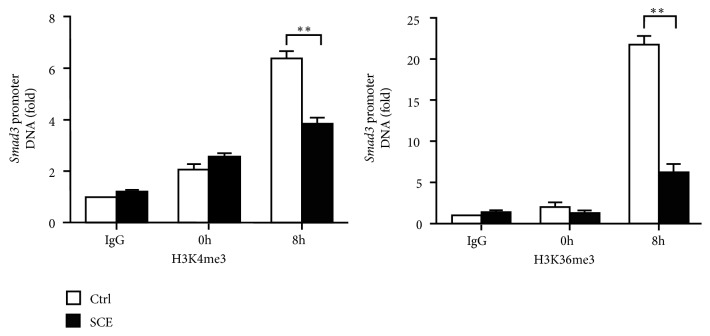
SCE decreases the levels of H3K4me3 and H3k36me3 at the* Smad3* promoter in CFs. ChIP-qPCR analysis of H3K4me3 or H3K36me3 level at the* Smad3* promoter in CFs pretreated with SCE (10 *μ*l/ml) followed by TGF-*β* (20 ng/ml) stimulation for the indicated times. IgG, control ChIP with immunoglobulin G. Data are from three independent experiments (mean ± SEM). ^*∗∗*^*P* < 0.01, Student's* t*-test.

**Figure 7:**
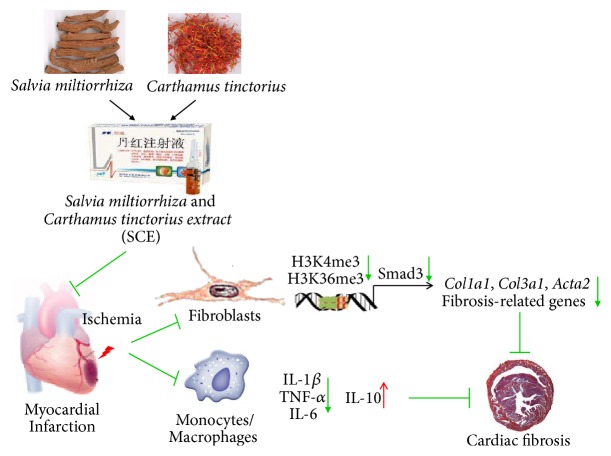
SCE attenuates myocardial fibrosis and adverse remodeling after MI by suppressing histone methylation of* Smad3* promoter and its transcription.

Next, we explored the mechanism underlying the decreased expression mediated by SCE. H3K4me3 and H3K36me3, two types of important histone methylation modifications at gene promoters, can promote gene transcription. Thus, we investigated whether SCE affected histone methylation modifications at the* Smad3* promoter. As shown in Figure 6, the SCE treatment significantly decreased the levels of H3K4me3 and H3k36me3 at the* Smad3* promoter in the TGF-*β*-stimulated CFs. Thus, SCE attenuates myocardial fibrosis and adverse remodeling after MI by suppressing histone methylation of the Smad3 promoter and Smad3 transcription (Figure 7).

## 4. Discussion

Our results showed that the administration of SCE attenuated ventricular remodeling by inhibiting myocardial fibrosis in mice 21 days after the ligation of LAD. In addition, we found that attenuation of SCE on fibrosis was associated with downregulation of TGF-*β*/Smad3 signaling and SCE reduced increased levels of H3K4me3 and H3k36me3 in the* Smad3* promoter region induced by TGF-*β* in CFs.

In a recently published study [20], reduction of infarct size and attenuation of adverse remodeling were observed in rats of MI after 4 weeks of administration of SCE. It was shown that the combination of decreased myocyte death, inhibited fibrotic cytokines, and increased VEGF-related angiogenesis contributed to the therapeutic effect. Our study confirmed similar effects of SCE. Moreover, we found that proinflammatory proteins were downregulated by SCE with the concurrent inhibition of profibrotic cytokines. The finding was consistent with several previous studies involving endothelial cells [21] and cardiomyocytes [19], and we focused on CFs, a key regulator of cardiac fibrosis. Inflammation, as a bridge between injury and healing, plays a pivotal role in pathophysiological processes after MI. Although inflammation acts to eliminate debris from damaged tissues and subsequently initiate reparative fibrosis, the excessive or prolonged inflammatory reaction appears to be detrimental in post-MI healing and remodeling, which is likely to lead to infarction expansion and even ventricular rupture [22]. The transformation of CFs into myofibroblasts can be regulated by various inflammatory cytokines, which are also secreted by myofibroblasts to amplify inflammation. Therefore, on one hand, inflammatory factors, such as IL-1 and TNF-*α*, activate CFs to proliferate and transform into myofibroblasts, showing the characteristics of fibrosis. On the other hand, the level of inflammation also reflects the degree of fibrotic response [23, 24]. Though it is unknown how inflammation regulates fibrotic processes, it is certain that the inhibition of inflammatory-fibrotic response in fibroblasts at a specific phase will have beneficial effects on post-MI remodeling. Taken together, results derived from current research implied that, when used from the initial phase to the maturation phase, SCE may be more effective for inhibiting adverse remodeling.

In this study, we further screened the proteins involved in the TGF-dependent pathway and found no significant changes in the protein expression that regulates cell proliferation, differentiation, and damage repair, whereas Smad3 in the SMAD family was inhibited by SCE uniquely. Myocardial fibrosis, which leads to adverse remodeling, has not been addressed explicitly in the current guidelines for the treatment of MI [25]. An abundance of data demonstrated that the TGF-*β*/Smad3 system is pivotal in fibrosis and postinfarction remodeling [7]. A recent study suggested that fibroblast-specific Smad3 signaling played a beneficial role in the infarct healing via mediating the formation of an organized scar, by restraining fibroblast proliferation, and by stimulating integrin-dependent fibroblast activation, fibroblast-derived collagen synthesis, and subsequent formation of well-aligned arrays of myofibroblasts in the infarct border zone [26]. Results from our research seemed inconsistent with the results in the above research. However, mice with global loss of Smad3 and LAD ligation showed the attenuated cardiac remodeling compared with wild-type mice [27]. A possible explanation for this contradiction might be that reparative and profibrotic fibroblast functions are mediated through distinct molecular pathways or are from diverse origins [28]. SCE, like other products derived from Chinese herbs, is a mixture of various ingredients. However, it is not clear which component of SCE selectively acts on Smad3 in our study. Salvianolic acid B, one of the main pharmacological active constituents of SCE, was reported to suppress TGF-*β*1-induced expressions of collagen I and Smad3 in primary hepatic stellate cells [29], which is the cellular resource for liver fibrogenesis. Therefore, it is reasonable to infer that salvianolic acid B may be the active effector in SCE acting on Smad3 to inhibit cardiac fibrosis after MI, which needs to be further investigated.

In the current research, the inhibition of SCE on histone methylation in the* Smad3* promoter induced by TGF-*β* in CFs suggested the underlying mechanism of fibrosis suppression. To our knowledge, the relationship between histone methylations of Smad3, both H3K4me3 and H3K36me3, and fibrosis has not yet been reported in heart disease but has been previously investigated, mainly in cancer [30] and fibrosis of other organs [31]. Compared to epigenetic research on cardiac fibrosis, like histone acetylation, fewer studies have been conducted on the effect of histone methylation. H3K4me3 and H3K36me3 typically are associated with transcription activation, while H3K9 and H3K27 methylations are linked with transcription repression [32]. Several studies have indicated that histone methylation is involved in cardiac fibrosis and heart failure. Pandya et al. identified a close link between H3K4me3 and the reactivation of the *β*-myosin heavy chain (*β*MHC) [33], which is regarded as a cornerstone in the field of histone methylation with heart failure. Liming Yu et al. reported that upregulation of the H3K4me3 level activated by Suv, Ez, and Trithorax domain 1 (SET1), a mammalian histone H3K4 trimethyltransferase, is essential for the expression of the ET-1 gene in endothelial cells and the formation of fibrotic proteins in mouse hearts induced by Ang-II [34]. A further study showed that endothelial-specific knockdown of either H3K4 methyltransferase complex or related regulators by shRNA attenuated Ang-II-induced cardiac hypertrophy and fibrosis [35]. Compared with these histone methylation studies in cardiac fibrosis, our research may be quite different in the animal model and target gene. In short, our findings suggested that epigenetic modulation of local H3K4me3 and H3K36me3 in the* Smad3* promoter may be a potential therapeutic target against heart failure after MI.

## 5. Conclusion

In summary, our data indicated that SCE reduced fibrosis after MI and improved heart function via inhibition of the TGF-*β*/Smad3 pathway. The data has also shown that SCE suppressed TGF-*β*-induced H3K4me3 and H3K36me3 at the* Smad3* promoter. Overall, our findings shed light on the potential of long-term clinical application of traditional Chinese medicine as supplementary in ischemic heart failure. Also, this study contributes to our understanding of epigenetic modification in ventricular remodeling and highlights the need to conduct more research in this field.

## Data Availability

The data used to support the findings of this study are available from the corresponding author upon request.

## Abbreviations

*α*-SMA: *α*-Smooth muscle actin
CFs: Cardiac fibroblasts
ChIP: Chromatin immunoprecipitation
H3K4me3: H3K4 trimethylation
H3K36me3: H3K36 trimethylation
LAD: Left anterior descending
MI: Myocardial infarction
SCE: * Salvia miltiorrhiza* and* Carthamus tinctorius* extract
Smad: Drosophila mothers against decapentaplegic protein.

## Supplementary Materials

Supplementary Materials Figure S1 in the Supplementary Materials provides additional results of SCE on TGF-*β* signaling pathway in heart tissue after MI. Figure S2 shows the effect of SCE at different concentrations on apoptosis of CFs. Figure S3 shows the expression of fibrosis-related genes in TGF-*β*-stimulated CFs upon SCE treatment. Supplemental Table 1 lists primers for detection of gene expression through RT-qPCR.
