# Supplementary material for: Salvia miltiorrhiza and Carthamus tinctorius Extract Prevents Cardiac Fibrosis and Dysfunction after Myocardial Infarction by Epigenetically Inhibiting Smad3 Expression
Source: Evid Based Complement Alternat Med. 2019 May 30;2019:6479136. doi: 10.1155/2019/6479136 (PMC6582873; doi:10.1155/2019/6479136)
Supplement: Supplementary Materials — Figure S1 in the Supplementary Materials provides additional results of SCE on TGF-β signaling pathway in heart tissue after MI. Figure S2 shows the effect of SCE at different concentrations on apoptosis of CFs. Figure S3 shows the expression of fibrosis-related genes in TGF-β-stimulated CFs upon SCE treatment. Supplemental Table 1 lists primers for detection of gene expression through RT-qPCR. [file 6479136.f1.pdf]

Supplementary material

***Salvia miltiorrhiza* and *Carthamus tinctorius* extract prevents cardiac fibrosis and dysfunction after myocardial infarction by epigenetically inhibiting Smad3 expression**

Jing Yang, Bo Wang, Na Li, Qingqing Zhou, Wenhui Zhou, Zhenzhen Zhan

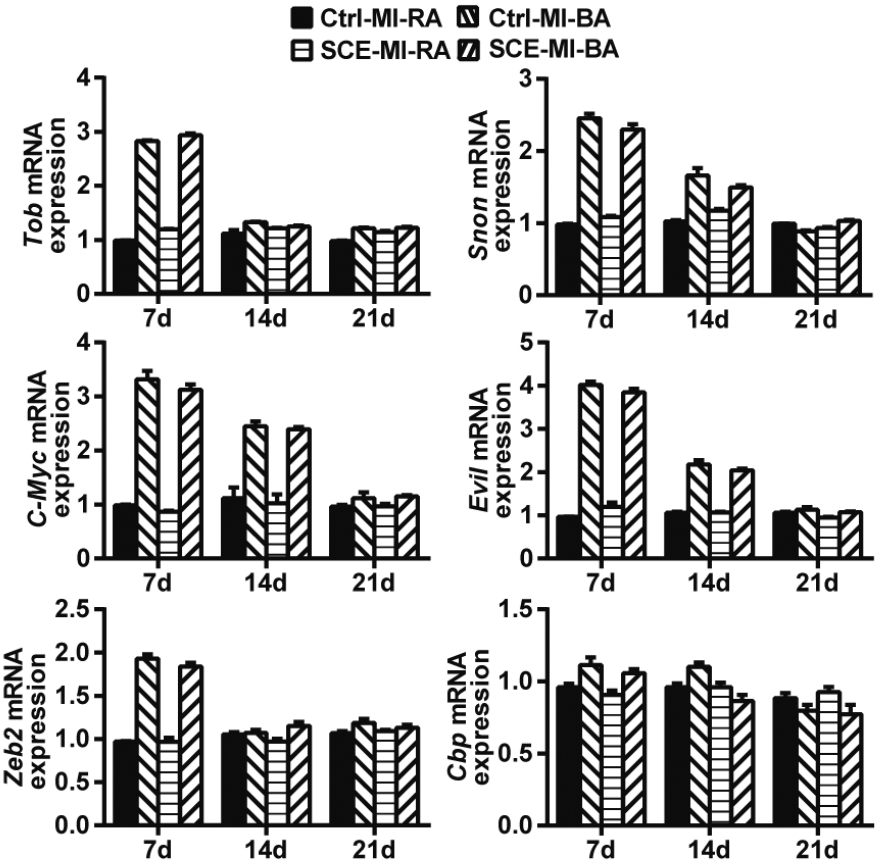

Figure S1. **SCE administration does not affect the expression level of some molecules in the TGF-β signaling pathway in heart tissue after MI.**

Mouse heart tissue isolated 7 d, 14 d and 21 d after MI were divided into border area (BA) and remote area (RA) for RNA extraction. Quantitative PCR was performed to analyze mRNA expression changes of *Tob*, *Snon*, *C-Myc*, *Evi1*, *Zeb2*, and *Cbp*, which are related to the TGF-β signaling pathway. No significant differences were found between the SCE administration group (SCE) and control group (Ctrl). Data are from three independent experiments (mean ± SEM).

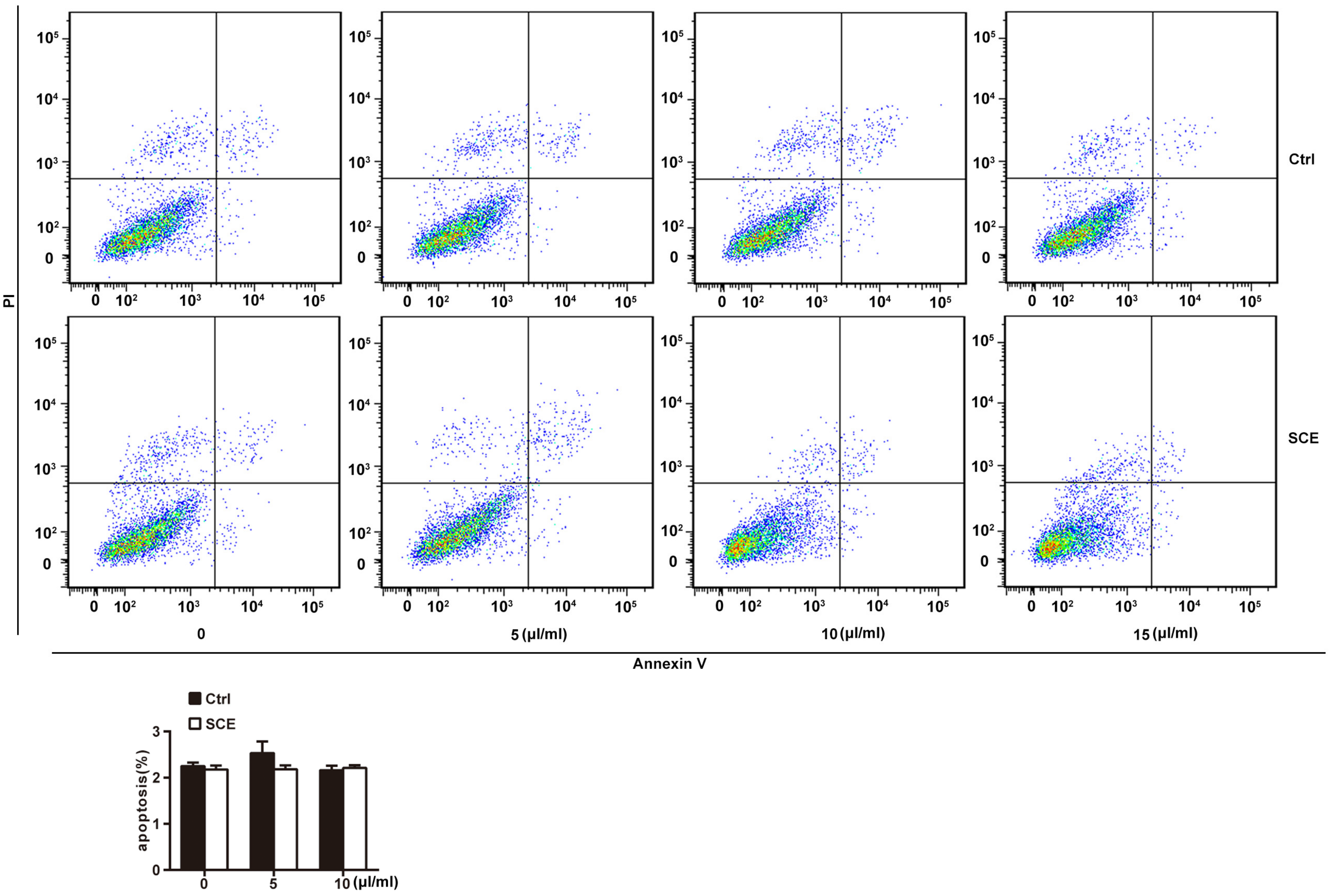

Figure S2. The treatment of SCE at different concentrations does not induce apoptosis of CFs.

Primary cardiac fibroblasts were separated from the neonatal mice for culture. The P1 cells were treated with serum-free medium for 24 h first and then were treated with PBS (Ctrl) or SCE at concentrations of 5 μl/ml, 10 μl/ml, and 15 μl/ml for 24 h, respectively. Trypsin-EDTA (2.5%) was then added to digest the SCE-pretreated cells. After 4 min of digestion, the P1 cells were labeled with annexin and PI for 10 min. Then, the difference in cell apoptosis rates between the two groups was analyzed by

flow cytometry. No significant difference in apoptosis was observed in cells with or without SCE administration.

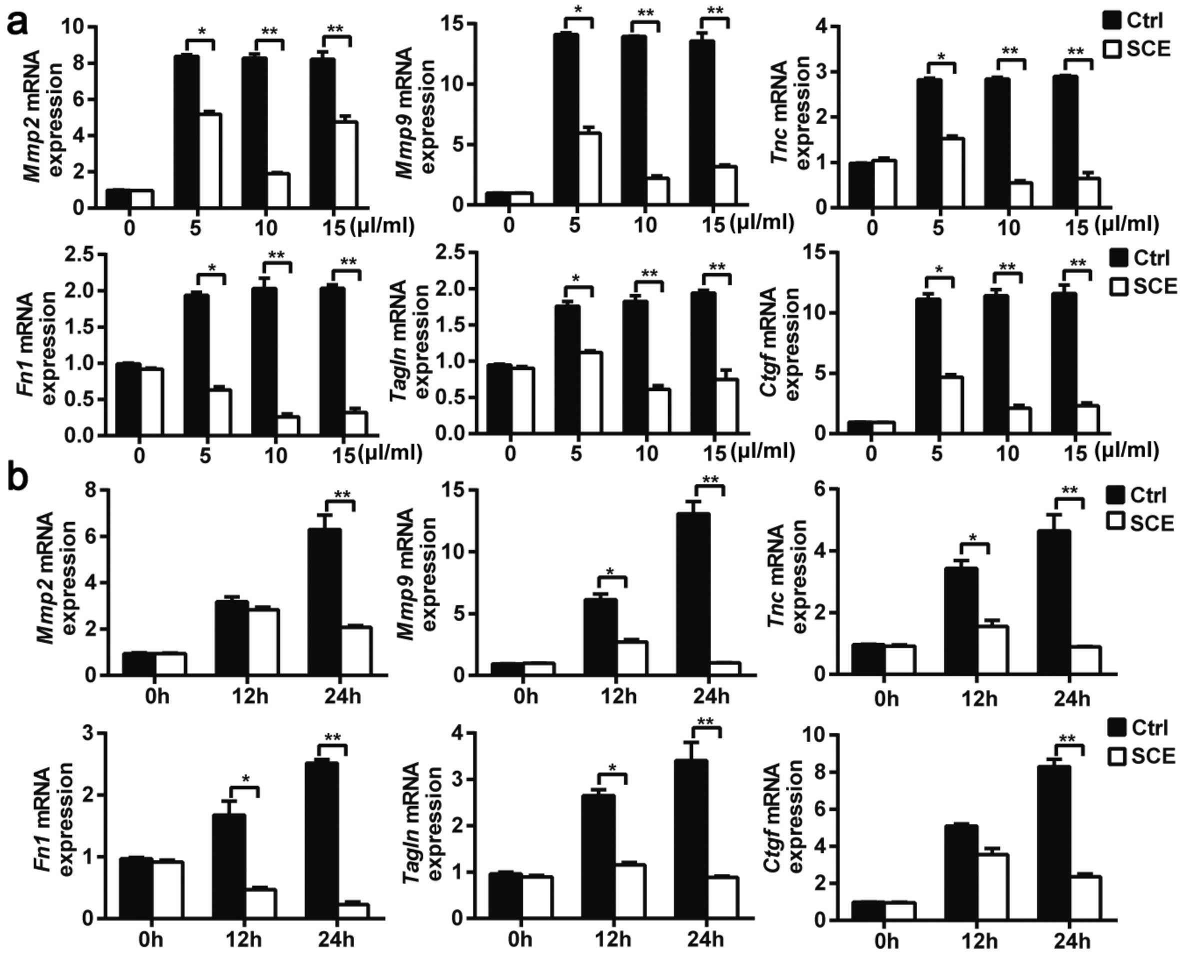

Figure S3. SCE treatment inhibits the expression of fibrosis-related genes in CFs stimulated with TGF-β.

(a) Primary cardiac fibroblasts isolated from the neonatal mice were treated with serum-free medium for 24 h. After pretreatment with PBS (Ctrl) or SCE at

concentrations of 5 μl/ml, 10 μl/ml, and 15 μl/ml for 30 min, respectively, TGF-β (20 ng/ml) was added to stimulate the pretreated cells for 24 h. After RNA extraction, RT-qPCR was performed to determine the expression level of gene mRNA. Expression of matrix metalloproteinase-2 (*Mmp2*), matrix metalloproteinase-9 (*Mmp9*), tenascin (*Tnc*), fibronectin1 (*Fn1*), transgelin (*Tagln*), and connective tissue growth factor (*Ctgf*) were significantly decreased in SCE group as compared with control group. (b) Primary cardiac fibroblasts from the neonatal mice were pretreated with PBS (Ctrl) or SCE (10 μl/ml) for 30 min, then stimulated with TGF-β (20 ng/ml) for 12 h or 24 h. After RNA extraction, RT-qPCR was performed to determine the expression level of gene mRNA. Expression of *Mmp2*, *Mmp9*, *Tnc*, *Fn1*, *Tagln*, and *Ctgf* were significantly decreased in the SCE group as compared with the control group. Data are from three independent experiments (mean ± SEM). $^{*}P < 0.05$; $^{**}P < 0.01$; Student's *t*-test.

Supplemental Table 1. Primers for detection of gene expression through RT-qPCR.

Genes		Primer sequence (5'……3')
Il1b	forward	GTTCCCATTAGACAACTGCACT
	reverse	CCACAGGTATTTTGTCGTTGCT
Il6	forward	AGACAAAGCCAGAGTCCTTCA
	reverse	CTTGGTCCTTAGCCACTCCT
Tnf	forward	GCATGGATCTCAAAGACAACCA
	reverse	ATGGGCTCATACCAGGGTTT
Il10	forward	GCTCTTACTGACTGGCATGAG
	reverse	CGCAGCTCTAGGAGCATGTG
Col1a1	forward	CGATGGATTCCCGTTCGAGT
	reverse	ATGACTGTCTTGCCCCAAGT
Col3a1	forward	TCCTAACCAAGGCTGCAAGA
	reverse	GGCTGGAAAGAAGTCTGAGGAA
Acta2	forward	GGCACCACTGAACCCTAAGG
	reverse	ACAATACCAGTTGTACGTCCAGA
Mmp2	forward	CGCTGCGCTTTTCTCGAATC
	reverse	CGGACCACTTGTCCTTCTCC
Mmp9	forward	AGTACTCTTCCTGTACCAGCG
	reverse	TCGAGGTAGCTATACAGCGG
GAPDH	forward	ATGTGTCCGTCGTGGATCTG
	reverse	AAGGAGTAAGAAACCCTGGACC
Smad2	forward	AAGCCATCACCACTCAGAATTG
	reverse	CACTGATCTACCGTATTTGCTGT
Smad3	forward	CACAGCCACCATGAATTACG
	reverse	TGGAGGTAGAACTGGCGTCT
Smad4	forward	ACACCAACAAGTAACGATGCC
	reverse	AGCCACCTGAAGTCGTCCA
Smad7	forward	GGGCTTTCAGATTCCCAACTT
	reverse	AGGGCTCTTGGACACAGTAGA
Msg1	forward	ACTAGCTCCTCTGGATCGACA
	reverse	GACCCAGTTTTGCATGGGC
Strap	forward	GCATCACGCCTTACGGCTA
	reverse	AATCCAGTCTCCTGTATCTCCC
Smurf1	forward	AGCATCAAGATCCGTCTGACA
	reverse	CCAGAGCCGTCCACAACAAT
Smurf2	forward	CCATTTGCTAAGGTGGTAGTTGA
	reverse	CAGGTCATAATGCTGATTCCACT
Tob	forward	ATATGAAGGGCACTGGTATCCT
	reverse	GGATGCCTGCTCGATCACG

Snon	forward	ACGCTGGCACAATTCCACTTA
	reverse	AGTCGGTGACATGCTCTCCT
C-Myc	forward	ATGCCCCTCAACGTGAACTTC
	reverse	GTCGCAGATGAAATAGGGCTG
Evi1	forward	AAGTAATGAGTGTGCCTATGGC
	reverse	AGTTGACTCTCGAAGCTCAAAC
Zeb2	forward	CCACGCAGTGAGCATCGAA
	reverse	CAGGTGGCAGGTCATTTTCTT
Cbp	forward	GGCTTCTCCGCGAATGACAA
	reverse	GTTTGGACGCAGCATCTGGA
Tnc	forward	ACGGCTACCACAGAAGCTG
	reverse	ATGGCTGTTGTTGCTATGGCA
Fn1	forward	CAAGACCATACCTGCCGAATG
	reverse	GTAGGTGACCCCTCTGGTAAG
Tagln	forward	CCAACAAGGGTCCATCCTACG
	reverse	ATCTGGGCGGCCTACATCA
Ctgf	forward	GGGCCTCTTCTGCGATTTC
	reverse	ATCCAGGCAAGTGCATTGGTA
